# A Reliability Study of Small, Portable, Easy-to-Use, and IMU-Based Sensors for Gait Assessment

**DOI:** 10.3390/s25216597

**Published:** 2025-10-26

**Authors:** Maciej Tomasz Kochman, Aleksandra Kielar, Marta Kasprzak, Wojciech Kasperek, Martin Dutko, Adam Vellender, Grzegorz Przysada, Mariusz Drużbicki

**Affiliations:** 1 Department of Clinical Physiotherapy in Musculoskeletal Disorders, Faculty of Health Sciences and Psychology, Institute of Physiotherapy, College Medicum, University of Rzeszów, Tadeusza Rejtana Avenue 16C, 35-310 Rzeszów, Poland; 2 Faculty of Medicine, College Medicum, University of Rzeszów, Tadeusza Rejtana Avenue 16C, 35-310 Rzeszów, Poland; 3 Rockfield Software Limited, Swansea SA1 8AS, UK; 4 Department of Mathematics, Aberystwyth University, Aberystwyth SY23 3BZ, UK

**Keywords:** gait, walking, sensors, wearable devices, reliability

## Abstract

The standard motion analysis systems are limited to laboratory settings; therefore, an individual’s gait may not be realistic, as it is removed from the day-to-day environment in which people ambulate. The modern world and advanced technologies have driven portable, affordable, and wearable sensors for real-world gait assessment that can be used outside the laboratory and during day-to-day activities. Wearable sensors offer a promising solution; however, despite that, the reliability of many wearable systems, especially under unsupervised and real-world-like conditions, remains insufficiently validated. This study aimed to establish intra- and inter-rater reliability of the inertial sensors as a tool used in gait analysis in a quasi-real-world environment. Ninety-eight healthy participants (52% females) aged 19–33 years took part in this reliability study. The research procedures included two separate measurements of gait analysis at participants’ preferred walking speed, conducted by two raters assessing selected spatiotemporal parameters. The reliability was calculated using intraclass correlation coefficients (ICC), and the bias was assessed using the Bland–Altman method. The analysis of intraclass correlation coefficients (ICC) revealed excellent, or near-excellent, reliability for walking speed, cadence, and stride length between raters (ICC = 0.932–0.941, 0.950–0.957, and 0.916–0.938, respectively) and between measurements (ICC = 0.916–0.928, 0.934–0.938, and 0.888–0.906, respectively). Bland–Altman plots confirmed minimal systematic bias for both inter- and intra-rater assessments, with differences in walking speed below 0.038 km/h, cadence below 0.283 steps/min, and stride length below 0.827 cm. The examined sensors are reliable tools for walking speed, cadence, and stride length in a quasi-real-world environment gait assessment. Future studies should include gait analysis involving random path and direction changes, turns, uneven or slippery surfaces, and natural environments. Additionally, research should consider individuals ambulating with various walking aids, or those with unilateral disorders, such as stroke.

## 1. Introduction

Walking is a spontaneous and natural physical activity among humans. It is also an extremely important health indicator, as gait disturbance is linked to various conditions such as sarcopenia or arthritis, but it also may predict falls, frailty, disability, dependence, or even mortality in older adults [1,2,3,4,5,6,7,8]. The basic unit to characterize the way of walking is a gait cycle (also known as a stride), which consists of two phases: the stance (approx. 60% of the gait cycle) and the swing (approx. 40% of the gait cycle) [9]. The gait cycle may be characterized by spatial and temporal parameters. Spatial parameters include the step length (the distance from one heel to another), step width (the distance between two heel strikes), and stride length (the distance between same-side heels), while the temporal parameters include the gait speed, the cadence (steps per minute), and the duration of step, stride, and each gait phase [8,10,11].

Various methods have been proposed by researchers to examine the gait and its abnormalities. One of them is a camera-based motion capture system, assessing gait in a controlled experimental environment [12]. During such assessment, the participants have several visual markers placed on specific parts of their bodies, and then they are asked to walk a certain distance, which is monitored by several cameras installed in the laboratory [13,14]. This method shows a high accuracy in determining gait abnormalities [14]. However, the standard motion analysis systems are limited to laboratory settings; therefore, an individual’s gait may not be realistic, as it is removed from the day-to-day environment in which people ambulate [14,15]. Also, as they require several cameras, they are quite difficult to install, calibrate, and operate [16]. Finally, such motion systems laboratories are costly and must be operated by professionally trained personnel [16,17]. Despite the accurate and reliable gait parameters data, such gait analyses are restricted to high-tech gait laboratories; therefore, the need arose for cheaper and portable methods, allowing simple, precise, and cost-effective gait assessment in various environments [16,18].

The modern world and the advanced technologies have been driven to portable, affordable, and wearable sensors for real-world gait assessment that can be used outside the laboratory and during day-to-day activities [19,20]. Wearable devices include accelerometers, magnetometers, and gyroscopes, combined as inertial measurement units (IMU) or pressure insoles, allowing us to collect and analyze kinematic, kinetic, and spatiotemporal gait parameters [20,21].

Previous studies have presented the use of several various wearable technologies analyzing out-of-lab gait parameters; however, some of them still lack the reliability assessment, and therefore, researchers recommend future studies to obtain the reliability of wearables-based measurements of gait quality [22,23]. Given the limitations of traditional gait analysis systems, such as high cost, limited accessibility, and artificial laboratory conditions, there is a growing need for reliable and portable alternatives that may be used in more natural settings. Wearable sensors offer a promising solution, allowing for real-time, out-of-lab gait assessment during daily activities. However, despite that, the reliability of many wearable systems, especially under unsupervised and real-world-like conditions, remains insufficiently validated. Encouraged by that, we conducted this study to establish intra- and inter-rater reliability of the PABLO^®^ Lower Extremity inertial sensors (Tyromotion GmbH, Graz, Austria) as a tool for gait analysis in a quasi-real-world environment—specifically, unsupervised conditions where participants were dressed normally and could autonomously choose their walking speed. To the best of the authors’ knowledge, such studies are missing in the relevant scientific literature.

## 2. Materials and Methods

### 2.1. Ethical Considerations

The protocol of this prospective study was approved by a local Bioethics Committee (approval number: 2024/02/008) on 12 February 2024, and all study procedures were conducted in compliance with the Declaration of Helsinki. Before the start of the study, all participants were informed about the details of the study, and all participants gave their written informed consent to participate in the study.

### 2.2. Eligibility Criteria

To be included in the study, all study participants had to meet the following eligibility criteria: informed consent; age above eighteen years; no lower limbs or spine injuries/surgeries in the past twelve months; no past medical histories that would be a possible contraindication to the walking or physical activity, or causing abnormal gait pattern; and not practicing sports professionally.

### 2.3. Sample Size Calculation

Before the start of the study, the minimum sample size was calculated using the following parameters: a 95% confidence interval, a 0.9 fraction size, and a 5% maximum error. Based on that, the minimal population required for this study was ninety-eight individuals.

### 2.4. Study Participants

**Figure 1:**
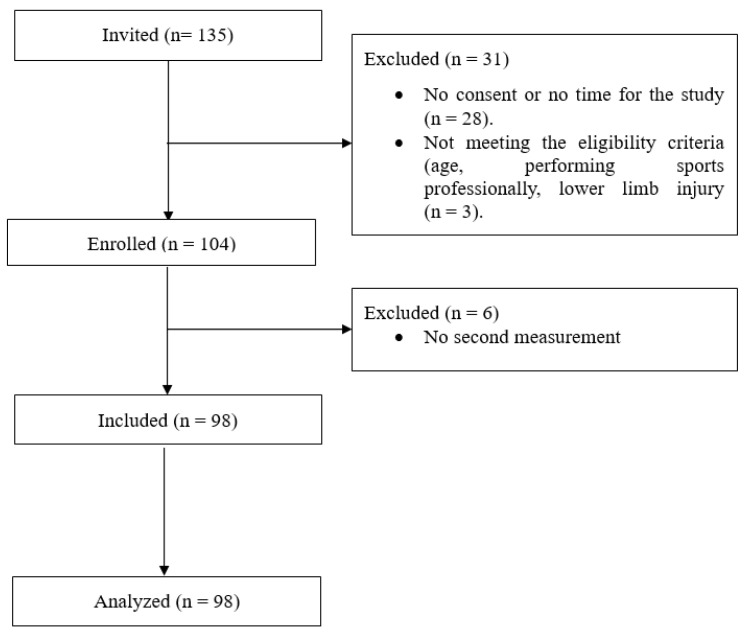
Study participants’ flowchart.

One hundred and thirty-five persons were invited to this study. As twenty-eight persons did not consent to participation in the study, and three persons did not meet the eligibility criteria, one hundred and four participants were recruited. Six participants did not perform the second measurement, and their results were excluded from the final analysis. Finally, we analyzed the data of ninety-eight participants. All participants were of Caucasian ethnicity. The study participant flowchart is presented in Figure 1.

The study group (n = 98) consisted of males (48%) and females (52%) aged 19–33 years (mean age 23.16), and the mean BMI of the group was 23.56. Detailed study group characteristics are shown in Table 1.

### 2.5. Research Procedures

This study was conducted in April 2024 in the Department of Physiotherapy, College of Medical Sciences, University of Rzeszów, Poland. The gait analysis was performed using the PABLO^®^ Lower Extremity inertial sensors (Tyromotion GmbH, Graz, Austria). The IMU data were recorded using the TyroS software (Tyromotion, Graz, Austria), which allowed us to export the raw gyroscope and accelerometer data. The dimensions of each sensor were 56 × 34 × 21 mm. These calibration-free sensors detected acceleration and angular rate, and transferred the data wirelessly via Bluetooth with a frequency of 110 Hz, with high accuracy for various walking speeds and abnormal gait patterns [24].

The research procedures included two separate gait analysis measurements conducted within two weeks by two raters, resulting in a total of four measurements for each participant. To clarify, we have labeled the two separate measurements as Exam 1—the first measurement—and Exam 2—the second measurement—conducted two weeks after Exam 1. The raters who performed the assessments are referred to as Rater 1 (M.Ka.) and Rater 2 (A.K.), indicating the first and second assessors, respectively.

**Figure 2:**
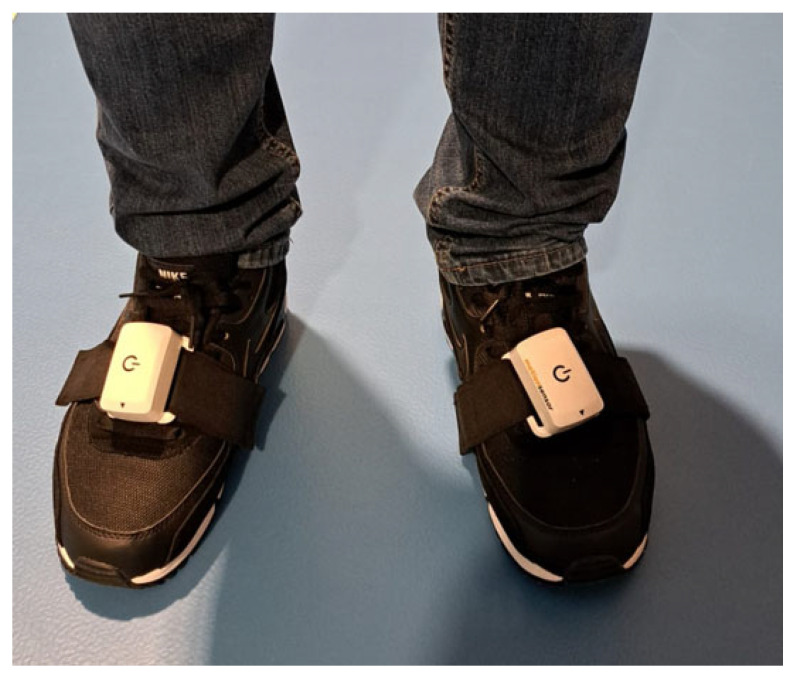
The placement of the sensors on the participants’ shoes.

In this study, we analyzed gait spatiotemporal parameters, such as walking speed (km/h), cadence (steps/minute), and stride length (cm). During the study, the participants were wearing clothes and shoes they typically wear to imitate real-life conditions. The inertial sensors were placed on the participants’ shoes with a dedicated Velcro strap. After each sensor placement, participants were asked whether the sensors felt comfortable and acceptable. Before each test, participants were also asked to walk at a comfortable speed to ensure they felt at ease. Then, the participants were asked to walk a designated 10 m linear distance along the corridor at their usual, self-selected walking speed, reflecting their typical ambulation in daily life. Except for that, no additional instructions were given to the participants. For each measurement, each rater put sensors on the participants’ feet, and after the measurement, they removed the sensors from the feet. The sensors’ placement is shown below, in Figure 2.

### 2.6. Statistical Analysis

The analysis of the data was performed in Statistica 13.3 (Statsoft, Kraków, Poland) and Python 3.11; plots were generated using Matplotlib 3.10.6. Descriptive statistics for study group characteristics (age, height, body weight, and BMI) were calculated and presented using mean, standard deviation (SD), median, and first and third quartiles, as well as minimum and maximum values.

The distribution of the data was assessed using the Shapiro–Wilk test. As all the data was normally distributed, parametric tests were used for further analysis. The differences between the measurements of walking speed, cadence, and stride length were calculated using the dependent samples *t*-test. The level of significance was assumed at *p* < 0.05.

The inter- and intra-rater reliability of the measurements were assessed using intraclass correlation coefficients (ICC). For intra-rater reliability, a single-measure, absolute-agreement, and two-way mixed-effects model (ICC(3,1)) was used. For inter-rater reliability, the data were calculated based on a mean-measure, absolute-agreement, and two-way random-effects model (ICC(2,k), with k = 2 raters). Ninety-five percent confidence intervals (95% CI) were calculated for all ICCs. The ICC agreement was established based on the following criteria, proposed by Koo et al.: below 0.50—poor; between 0.50 and 0.75—moderate; between 0.75 and 0.90—good; above 0.90—excellent. The variability of the data was summarized using the mean and standard deviation (SD), the mean difference and its standard deviation (SD), coefficients of variation (CV), and standard error of measurement (SEM) [25,26]. For each outcome (walking speed, cadence, stride length), the coefficient of variation (CV) was computed as the between-participant standard deviation for a single measurement, divided by the corresponding group mean, and expressed as a percentage: CV(%) = 100 × SD/Mean. CV therefore quantifies between-participant variability for each rater × exam condition. The standard error of measurement (SEM) for each inter- or intra-rater comparison was calculated from the standard deviation of the paired differences between the two repeated measurements as SEM = SD_diff_/√2. SEM therefore reflects within-participant measurement error for the specified comparison (e.g., Rater 1 vs. Rater 2 in Exam I; Exam I vs. Exam II for Rater 1).

In tables, D denotes the mean paired difference between the two measurements being compared (significance tested with a paired *t*-test); SD_diff_ denotes the standard deviation of those paired differences.

The Bland–Altman method was used to assess the bias between the two means of the measurements of the same rater (measurement I vs. measurement II), or between raters (measurement I vs. measurement I or measurement II vs. measurement II). In the Bland–Altman plot, the Y axis presents the difference between the measurements, while the X axis is their average. The limits of agreement (LoA) were calculated based on the values of the mean difference and the standard deviation (SD) of the differences, according to the formula: LoA = ±1.96 × SD. In the plot, 95% of all results should be located within the upper and lower LoA. The bias was assessed by using the mean difference and calculating its 95% confidence interval (CI). If the line of equity (the mean difference at the value of zero) is located within the 95% CI of the mean difference, then no systematic difference is considered to exist, and the mean difference can be assumed to be equal to zero [27,28].

## 3. Results

### 3.1. Reliability Results for Walking Speed (km/h)

The analysis of the ICC revealed an excellent agreement between raters and exams. The differences between all measurements were below 0.04 km/h and were insignificant. The ICC values for inter-rater reliability ranged from 0.932 to 0.941, while for intra-rater reliability, they were slightly smaller and ranged from 0.916 to 0.928 (Table 2). Inter-rater comparisons report D, SD_diff_, and SEM for Rater 1–Rater 2 within the same exam (Exam I and Exam II). Intra-rater comparisons report D, SD_diff_, and SEM for Exam II–Exam I within the same rater (Rater 1 and Rater 2). ICCs are reported with 95% CIs: inter-rater as ICC(2,k) with k = 2, and intra-rater as ICC(3,1), both with absolute agreement.

The Bland–Altman analysis revealed that the bias between the walking speed measurements—both inter- and intra-rater—was minimal, as the line of equality fell within the 95% confidence interval (CI) of the mean difference value in all plots. Additionally, most of the data points were located within the limits of agreement (LoA).

The inter-rater bias for walking speed ranged from 0.038 km/h (95% CI: −0.024 to 0.099; LoA: −0.570 to 0.646) in Exam I to 0.021 (95% CI: −0.041 to 0.084; LoA: −0.599 to 0.641) in Exam II. The intra-rater bias for walking speed ranged from −0.007 km/h (95% CI: −0.061 to 0.047; LoA: −0.538 to 0.524) for Rater 1 to −0.023 km/h (95% CI: −0.070 to 0.023; LoA: −0.487 to 0.440) for Rater 2.

**Figure 3:**
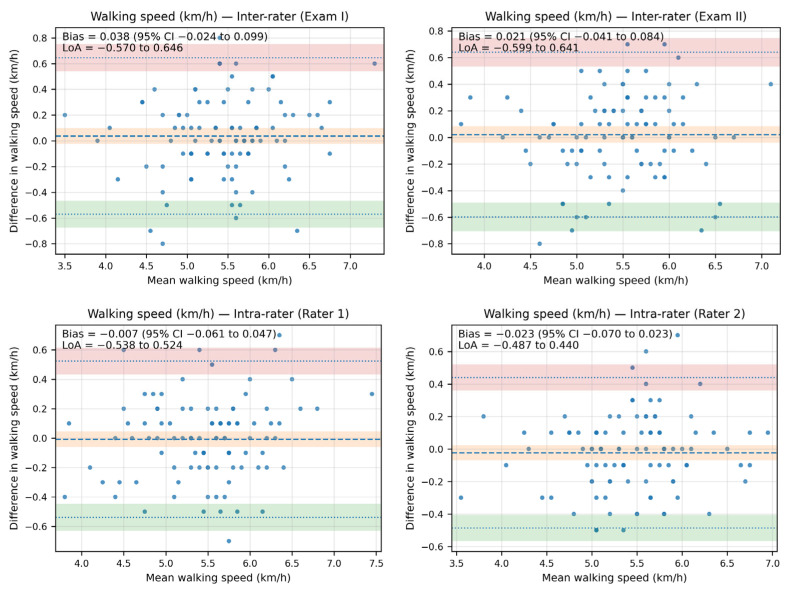
The Bland–Altman plots showing agreement between the measurements for walking speed (km/h).

The Bland–Altman plots illustrating agreement in walking speed measurements are presented in Figure 3.

### 3.2. Reliability Results for Cadence (Steps/min)

In cadence, the differences between the measurements were slightly higher compared to walking speed and were below 0.28 steps/minute. However, they were also insignificant. The ICC values were higher, and for inter-rater reliability, ranged from 0.950 to 0.957. For intra-rater reliability, the ICC values were lower and ranged from 0.934 to 0.938. In all the above analyses, the agreement between the measurements was excellent (Table 3). Inter-rater comparisons report D, SD_diff_, and SEM for Rater 1−Rater 2 within the same exam (Exam I and Exam II). Intra-rater comparisons report D, SD_diff_, and SEM for Exam II−Exam I within the same rater (Rater 1 and Rater 2). ICCs are reported with 95% CIs: inter-rater as ICC(2,k) with k = 2, and intra-rater as ICC(3,1), both with absolute agreement.

The Bland–Altman analysis revealed that the bias between the cadence measurements—both inter- and intra-rater—was minimal, as the line of equality fell within the 95% confidence interval (CI) of the mean difference value in all plots. Additionally, most of the data points were located within the limits of agreement (LoA).

The inter-rater bias for cadence ranged from 0.206 steps/minute (95% CI: −0.495 to 0.907; LoA from −6.730 to 7.143) in Exam I to 0.283 steps/minute (95% CI: −0.449 to 1.014; LoA: −6.957 to 7.522) in Exam II. The intra-rater bias for cadence ranged from −0.194 steps/minute (95% CI: −0.820 to 0.432; LoA: −6.388 to 6.00) for Rater 1 to −0.117 steps/minute (95% CI: −0.702 to 0.468; LoA: −5.910 to 5.675) for Rater 2.

**Figure 4:**
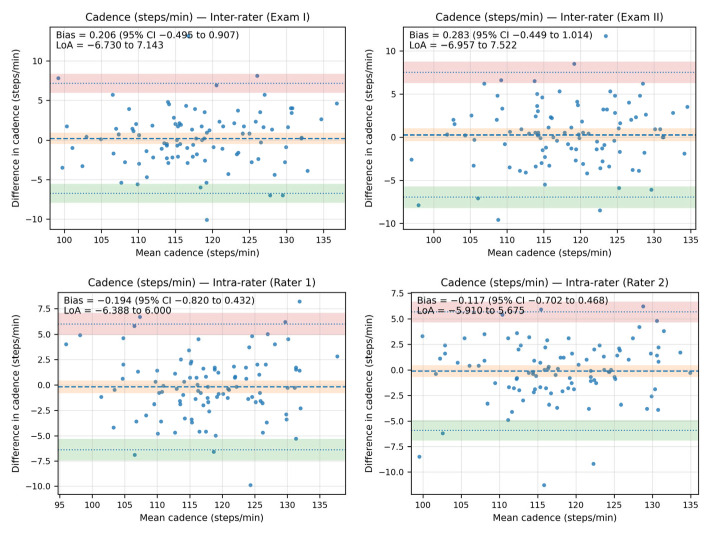
The Bland–Altman plots showing agreement between the measurements for cadence (steps/minute).

The Bland–Altman plots showing agreement between the measurements for cadence are shown in Figure 4.

### 3.3. Reliability Results for Stride Length (cm)

The analysis of the ICC revealed an excellent agreement between raters, and an almost excellent agreement between exams for stride length. The differences between all measurements were below 0.83 cm and were insignificant. The ICC values for inter-rater reliability ranged from 0.916 to 0.938, while for intra-rater reliability, they were smaller and ranged from 0.888 to 0.906 (Table 4). Inter-rater comparisons report D, SD_diff_, and SEM for Rater 1−Rater 2 within the same exam (Exam I and Exam II). Intra-rater comparisons report D, SD_diff_, and SEM for Exam II−Exam I within the same rater (Rater 1 and Rater 2). ICCs are reported with 95% CIs: inter-rater as ICC(2,k) with k = 2, and intra-rater as ICC(3,1), both with absolute agreement.

The Bland–Altman analysis revealed that the bias between the stride-length measurements—both inter- and intra-rater—was minimal, as the line of equality fell within the 95% confidence interval (CI) of the mean difference value in all plots. Additionally, most of the data points were located within the limits of agreement (LoA).

The inter-rater bias for stride length ranged from 0.724 cm (95% CI: −0.798 to 2.247; LoA from −14.347 to 15.796) in Exam I to 0.163 cm (95% CI: −1.089 to 1.416; LoA: −12.238 to 12.565) in Exam II. The intra-rater bias for stride length ranged from −0.265 cm (95% CI: −1.571 to 1.040; LoA: −13.192 to 12.661) for Rater 1 to −0.827 (95% CI: −1.931 to 0.278; LoA: −11.763 to 10.110) for Rater 2.

**Figure 5:**
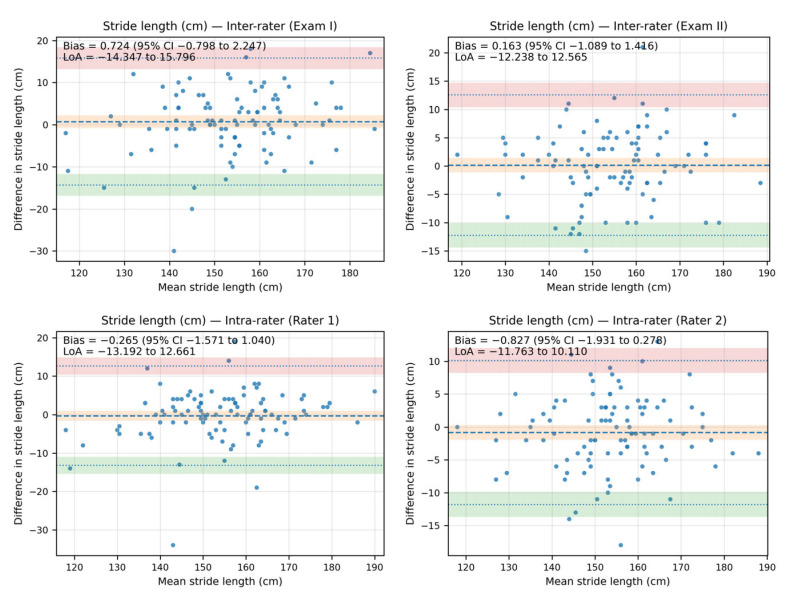
The Bland–Altman plots showing agreement between the measurements for stride length (cm).

The Bland–Altman plots showing agreement between the measurements for stride length are shown in Figure 5.

## 4. Discussion

In this study, we attempted to establish intra- and inter-rater reliability of the PABLO^®^ Lower Extremity inertial sensors in selected gait analysis parameters in a quasi-real-world environment. Our results showed that these sensors are reliable tools to measure walking speed, cadence, and stride in a quasi-real-world environment. Gait speed is a common measure to identify the individuals who are at risk of falling, and both low and high gait speed is linked to falls, as higher gait speed is related to higher activity levels and outdoor falls, while lower gait speed is related to deteriorated health, worse functional status and indoor falls [29]. Cadence is also a critical gait parameter, as it may also provide valuable substantial insight into the risk of falls, especially in the elderly, while monitoring free-living environment gait cadence. Urbanek et al. observed that across the community-dwelling older adults, higher cadence with every ten steps per minute was related to a 13.2% lower fall rate. In the case of higher-functioning older adults, higher cadence with every ten steps per minute was related to a 27.7% lower fall rate [30]. On the other hand, stride length gives additional biomechanical data, as it provides more in-depth analyses of gait biomechanics [31]. The pooled data with 687 participants indicated that individuals with lower back pain had shorter stride lengths when walking at the preferred speed [32]. Also, the results of the meta-analysis by Bytyçi and Henei support the significant value of stride length for predicting life-threatening clinical events in older adults. A stride length shorter than 0.64 m accurately predicts clinical events over other gait measures [33].

Our results are consistent with a previous study [34], assessing the measurement accuracy of a gait analysis method based on these sensors in the acquisition of the foot position and angle trajectories of the foot in the sagittal, frontal, and transversal plane over the entire gait cycle on the treadmill, at set speeds ranging from 1.5 km/h to 5 km/h. In our study, we decided to assess gait parameters at the participants’ preferred speed as they ambulate in real life. Nonetheless, according to Chaparro-Cárdenas et al., there is still much work to be undertaken in motion control to generate trajectories that closely resemble natural human movements [35]. This may include conditions such as walking along random paths and routes with direction changes and turns, leading to frequent variations in speed and step length, as well as uneven or slippery surfaces.

Our study has some limitations. First of all, we included healthy and young adult participants only. It would be more beneficial to include older adults or individuals across relevant conditions with pathological gait patterns, especially with unilateral disorders, such as stroke survivors, or people ambulating with various walking aids, such as sticks, in their natural environment, such as their home. Another limitation is that the participants were ambulating in a flat indoor space, which could facilitate a better drift correction compared to slippery or uneven surfaces in natural and real-world conditions. We also acknowledge that the method of sensor attachment (e.g., the tightness of the Velcro strap fixation) and variations in the types of footwear worn by participants could potentially influence the measurement results. Another limitation is that we did not compare IMU-driven gait parameters to gold-standard motion capture systems, which may affect the generalizability and clinical relevance of our findings. The strength of this study is a sample size of 98 individuals, which is considerable according to similar studies that include mainly less than 30 participants [21,36].

## 5. Conclusions

The findings obtained in this study showed excellent or near-excellent inter- and intra-reliability of PABLO^®^ Lower Extremity inertial sensors in evaluating walking speed, cadence, and stride length in a quasi-real-world environment. Future studies should further investigate these sensors in ‘true’ real-world conditions, such as random paths, routes with direction changes and turns leading to frequent variations in speed and step length, uneven or slippery surfaces, and both home and external environments. Additionally, research should consider the elderly ambulating with walking aids, or patients with unilateral disorders, such as stroke.

## Figures and Tables

**Table 1:** Study group characteristics.

n = 98							
Variable	Mean	Median	Min	Max	Q1	Q3	SD
Age	23.16	23	19	33	22	24	2.56
Height	173.19	173	148	195	165	180	9.31
Body weight	71.21	69	46	120	60	80	15.89
BMI	23.56	22.86	17.6	37.04	20.68	25.44	3.92

Abbreviations. Q1: first quartile; Q3: third quartile; SD: standard deviation; BMI: Body Mass Index.

**Table 2:** Reliability results for walking speed measurements.

Outcome Measure	Rater	Exam	Mean	SD *	D	SD_diff_	CV	SEM	ICC (95% CI)
	**Inter-rater reliability**								
**Walking speed (km/h)**	1	I	5.47	0.68	0.04	0.31	12.40	0.23	0.941 (0.913–0.960)
	2	I	5.43	0.64			11.74	0.22	
	1	II	5.47	0.64	0.02	0.32	11.61	0.22	0.932 (0.900–0.954)
	2	II	5.45	0.61			11.21	0.21	
	**Intra-rater reliability**								
	1	I	5.47	0.68	0.00	0.27	12.40	0.19	0.916 (0.876–0.942)
	1	II	5.47	0.64			11.61	0.18	
	2	I	5.43	0.64	0.02	0.24	11.74	0.17	0.928 (0.895–0.951)
	2	II	5.45	0.61			11.21	0.16	

Abbreviations. SD * (between-participant): standard deviation for the single listed measurement; D: mean paired difference (sign as defined in the caption); SD_diff_: standard deviation of paired differences; CV(%) = 100 × SD/Mean for the single listed measurement; SEM = SD_diff_/√2 for the stated comparison; ICC: intraclass correlation coefficient (95% CI).

**Table 3:** Reliability results for cadence measurements.

Outcome Measure	Rater	Exam	Mean	SD *	D	SD_diff_	CV	SEM	ICC (95% CI)
	**Inter-rater reliability**								
**Cadence (steps/minute)**	1	I	118.06	8.74	0.21	3.54	7.40	2.31	0.957 (0.936–0.971)
	2	I	117.85	8.58			7.28	2.27	
	1	II	118.25	8.63	0.28	3.69	7.30	2.73	0.950 (0.926–0.966)
	2	II	117.97	8.26			7.00	2.61	
	**Intra-rater reliability**								
	1	I	118.06	8.74	0.19	3.16	7.40	2.31	0.934 (0.903–0.955)
	1	II	118.25	8.63			7.30	2.28	
	2	I	117.85	8.58	0.12	2.96	7.28	2.10	0.938 (0.909–0.958)
	2	II	117.97	8.26			7.00	2.02	

Abbreviations. SD * (between-participant): standard deviation for the single listed measurement; D: mean paired difference (sign as defined in the caption); SD_diff_: standard deviation of paired differences; CV(%) = 100 × SD/Mean for the single listed measurement; SEM = SD_diff_/√2 for the stated comparison; ICC: intraclass correlation coefficient (95% CI).

**Table 4:** Reliability results for stride-length measurements.

Outcome Measure	Rater	Exam	Mean	SD *	D	SD_diff_	CV	SEM	ICC (95% CI)
	**Inter-rater reliability**								
**Stride length (cm)**	1	I	154.02	14.56	0.72	7.69	9.46	5.83	0.916 (0.877–0.943)
	2	I	153.30	13.01			8.48	5.20	
	1	II	154.29	13.24	0.16	6.33	8.58	4.59	0.938 (0.908–0.958)
	2	II	154.12	12.74			8.27	4.41	
	**Intra-rater reliability**								
	1	I	154.02	14.56	0.27	6.60	9.46	4.83	0.888 (0.837–0.923)
	1	II	154.29	13.24			8.58	4.39	
	2	I	153.30	13.01	0.83	5.58	8.48	4.11	0.906 (0.863–0.936)
	2	II	154.12	12.74			8.27	4.03	

Abbreviations. SD * (between-participant): standard deviation for the single listed measurement; D: mean paired difference (sign as defined in the caption); SD_diff_: standard deviation of paired differences; CV(%) = 100 × SD/Mean for the single listed measurement; SEM = SD_diff_/√2 for the stated comparison; ICC: intraclass correlation coefficient (95% CI).

## Data Availability

The datasets used and analyzed during the current study are available from the corresponding author upon reasonable request.
